# Crotoxin induces cytotoxic effects in human malignant melanoma cells in both native and detoxified forms

**DOI:** 10.3389/fphar.2024.1425446

**Published:** 2024-07-24

**Authors:** Tamires Cunha Almeida, Karina Cristina Giannotti, Lorena Morais Ribeiro Silva, Rafael Marques-Porto, Carlos DeOcesano-Pereira, Lauren Camargo, Ana Marisa Chudzinski-Tavassi, Paul Reid, Gisele Picolo

**Affiliations:** ^1^ Laboratory of Pain and Signaling, Butantan Institute, Sao Paulo, Brazil; ^2^ Laboratory of Development and Innovation, Butantan Institute, Sao Paulo, Brazil; ^3^ Centre of Excellence in New Target Discovering (CENTD), Butantan Institute, Sao Paulo, Brazil; ^4^ Celtic Biotech Ltd., Dublin, Ireland

**Keywords:** antitumoral activity, crotoxin, cytotoxicity, detoxified crotoxin, melanoma

## Abstract

**Introduction:** Melanoma, a highly aggressive skin cancer originating in melanocytes, poses a significant threat due to its metastatic potential. While progress has been made in treating melanoma with targeted therapies and immunotherapies, challenges persist. Crotoxin (CTX), the principal toxin in *Crotalus durissus terrificus* snake venom, exhibits various biological activities, including anti-tumoral effects across multiple cancers. However, its clinical use is limited by toxicity. Thus, exploring alternatives to mitigate adverse effects is crucial.

**Methods and Results:** This study investigates the antitumoral potential of CTX in its native and in a detoxified form, in melanoma cells. Firstly, we demonstrated that detoxified CTX presented reduced phospholipase activity. Both forms proved to be more cytotoxic to SK-MEL-28 and MeWo melanoma cells than non-tumoral cells. In SK-MEL-28 cells, where cytotoxic effects were more pronounced, native and detoxified CTX induced increased necrosis and apoptosis rates. We also confirmed the apoptosis death demonstrated by the activation of caspase-3 and 7, and the formation of apoptotic bodies. Furthermore, both CTX caused cell cycle arrest at the G2/M phase, interfering with melanoma cell proliferation. Cell migration and invasion were also suppressed by both CTX. These results confirm the antitumoral potential of CTX.

**Discussion:** The maintenance of the antiproliferative effects in the detoxified version, with reduced enzymatic activity often liked to harm effects, supports further studies to identify active parts of the molecule responsible for the interesting effects without causing substantial toxic events, contributing to the future use of CTX-derived drugs with safety and efficacy.

## 1 Introduction

Melanoma, constituting just 1% of skin cancer cases, holds the highest mortality rate among skin cancer types, responsible for 80% of related deaths (Saginala et al., 2021). The advancement of targeted therapies like tyrosine kinase inhibitors (Chapman et al., 2011; Hauschild et al., 2012) and MEK inhibitors (Flaherty et al., 2012), alongside immunotherapies (Schadendorf et al., 2015; Eggermont et al., 2018), has notably enhanced treatment efficacy and patient survival rates. However, the persistence of treatment resistance and associated toxicities presents a significant hurdle (Ernst and Giubellino, 2022). Notably, immunotherapy’s application has given rise to a novel spectrum of immune-related toxicities, impacting diverse organs, and leading to severe and enduring adverse consequences (Ralli et al., 2020).

Snake venoms stand as a valuable repository of compounds holding immense potential for pharmacological applications (Mohamed Abd et al., 2019). Crotoxin (CTX) is the main compound of *Crotalus durissus terrificus* venom, composed by two subunits; the acid subunit CA, also known as crotapotin, and the basic subunit phospholipase, also called CB, and presents various biological activities, including analgesic (Sant’Anna et al., 2019), immunomodulatory (Teixeira et al., 2020), antimicrobial (Canelli et al., 2020), and antiviral (Shimizu et al., 2017) effects. In the realm of antitumoral effects, CTX stands out for its efficacy against various tumor types. It achieves this through a multifaceted approach, inhibiting cell viability, reducing clonogenic capacity, causing cell cycle arrest, promoting apoptosis, and even impeding angiogenesis and *in vivo* tumor growth (Yan et al., 2007; Ye et al., 2011; He et al., 2012; Han et al., 2014; Brigatte et al., 2016; Muller et al., 2018; Almeida et al., 2021).

However, the translation potential of CTX to the clinical setting has encountered challenges. A phase I clinical trial explored CTX administration in patients with solid tumors unresponsive to standard therapies (Cura et al., 2002). Encouragingly, disease progression was notably slowed. Yet, the occurrence of neurotoxic effects, including diplopia, strabismus, palpebral ptosis, incoordination, and nystagmus, tempered the optimism (Cura et al., 2002). Recent developments in a phase I study demonstrated that a carefully managed dose escalation strategy could mitigate CTX adverse effects. However, this approach necessitates specialized infusion pumps, posing logistical difficulties across diverse oncology centers (Medioni et al., 2017).

Given this context, there is a clear call to explore alternatives that can alleviate neurotoxic impact of CTX, paving the way for its safer and more straightforward clinical use. Thus, our study takes on this challenge, investigating the antitumoral potential of both the native and detoxified forms of CTX against cutaneous melanoma cells, concurrently unraveling their mechanisms of action.

## 2 Methods

### 2.1 Crotoxin

Total crotoxin was purified from the crude venom of *C. durissus terrificus* snakes using the methodology outlined in a prior study (Wang et al., 2012). The purity was determined by high pressure liquid chromatography. The detoxification of CTX was performed by autoclaving (1 mg/mL in 0.9% saline for a 1 h cycle at 121°C).

### 2.2 Phospholipase activity

The phospholipase activity was determined using 4-nitro-3-octanoyloxy-benzoic acid (4N3OBA) (Life Sciences) as substrate. In a 96-well plate, the substrate 4N3OBA in buffer (Tris/HCL 10 mM, CaCl2 10 mM, and NaCl 100 mM, pH 7.8) and the native or detoxified form of CTX were added. The plate was incubated for 40 min at 37°C, and the absorbance was subsequently measured at 425 nm. Tests were conducted in triplicate.

### 2.3 Cell lines

SK-MEL-28 (HTB-72, ATCC) and MeWo (HTB-65, ATCC) human melanoma cells were cultured in DMEM/F12 supplemented with 10% fetal bovine serum, 100 U/mL penicillin G, and 100 U/mL streptomycin. Human lung fibroblasts MRC-5 (CCL-171, ATCC) cells were grown in high glucose DMEM supplemented with 5% fetal bovine serum, 100 U/mL penicillin G, and 100 U/mL streptomycin. All cells were maintained at 37°C in a humidified 5% CO2 atmosphere. MRC-5 non-tumoral fibroblasts were kindly provided by Dr. Glenda Nicioli da Silva from Universidade Federal de Ouro Preto (Minas Gerais, Brazil) and MeWo cell were kindly donated by Dr. Durvanei Augusto Maria from Butantan Institute (Sao Paulo, Brazil).

### 2.4 Cytotoxicity

Cells were seeded in 96-well plates at a density of 1 × 10^4^ cells per well. After a 24-h incubation period, cells were treated with native CTX (12.5–100 μg/mL) or detoxified CTX (12.5–100 μg/mL) for 24, 48, and 72 h in a humidified atmosphere of 5% CO2. The concentrations were defined based on previous studies using CTX (Yan et al., 2007; Han et al., 2014). Cells treated with culture medium were used as negative control (untreated cells) and cells treated with paclitaxel (85 μg/mL) were used as positive control. Paclitaxel is one of the most widely used antineoplastic agents, which primary mechanism of action is based on the stabilization of microtubules, blocking cell cycle progression and leading cell to death (Alqahtani et al., 2019). After treatment, the culture medium was removed, MTT solution (1 mg/mL) was added to each well. The plates were then incubated at 37°C for 3 h. Subsequently, 100 μL of DMSO was added to the wells, homogenized, and the absorbance was measured at 540 nm. The results were expressed as a percentage (%) of viable cells, with untreated cells considered 100% viable for reference. Tests were conducted in triplicate.

### 2.5 Apoptosis and necrosis assay

Apoptosis and necrosis rates were assessed using the FITC Annexin V/Dead Cell Apoptosis Kit (Thermo Fisher Scientific). SK-MEL-28 cells were initially seeded in 6-well plates at a density of 3.5 × 10^5^ cells per plate. After 24 h, cells were treated with native (12.5 μg/mL) or detoxified CTX (50 μg/mL) for 24 h. Cells treated with culture medium were used as negative control (untreated cells). Following treatment, cells were carefully collected, washed with PBS, and labeled with propidium iodide and annexin V according to the instructions provided in the kit. Subsequently, the labeled cells were analyzed using a flow cytometer (Becton–Dickinson FACS Canto II), with data from 10,000 cells collected for each sample. The experiments were conducted in triplicate. Unstained cells were classified as alive, cells stained only by annexin V were classified as early apoptotic, cells stained by both annexin V and propidium iodide were classified as late apoptotic, and cells stained only by propidium iodide were classified as necrotic. The results were expressed as percentage (%) of cells classified as alive, early apoptotic, late apoptotic, or necrotic. FlowJo v.10 software was employed for the analysis of the collected data.

### 2.6 Detection of caspases-3/7 activation by fluorescence-imaging

The detection of activated caspases 3 and 7 was assessed using the CellEvent Caspase-3/7 Green Detection Reagent (C10423, Thermo Fisher Scientific). Apoptotic cells with activated caspase-3/7 emit a bright green, while cells lacking activated caspase-3/7 exhibit minimal fluorescent signals (background). SK-MEL-28 cells were initially seeded in black Advanced TC 96-well microplates (Greiner Bio-One) at a density of 9 × 10^3^ cells per well. After 24 h of incubation, cells were treated with native (12.5 μg/mL) or detoxified CTX (50 μg/mL) for 24 h. Cells treated with culture medium were used as negative control (untreated cells). Staurosporine, a protein kinase inhibitor, is an inductor of apoptosis (Manns et al., 2011) and was used as a positive control (2.3 μg/mL). Following the treatment period, the culture medium was removed and the detection reagent was added as per the manufacturer’s recommendations. The cells were then incubated at 37°C for 30 min. Nuclei of the cells were stained with Hoechst 33,342 dye (62,249, Thermo Fisher Scientific). Plates were subjected to high-content imaging analysis, also known as High-Content Screening, HCS, on MetaXpress High-Content Image Acquisition & Analysis Software (Molecular Devices). The HCS was used to acquire 16 images per well at ×20 magnification. Cell quantification based on images was performed according to the module “Multi-Wave Cell Scoring”. Tests were conducted in triplicate and the quantitative data shown represent the fluorescence intensity (%) of caspases activity relative to the negative control.

### 2.7 Morphological analysis

SK-MEL-28 cells were initially plated onto 50-mm coverslips placed in a 24-well plate, with each well containing 9 × 10^3^ cells. These cells were allowed to incubate for 24 h. Subsequently, cells were treated with native (12.5 μg/mL) or detoxified CTX (50 μg/mL) for 24 h. Cells treated with culture medium were used as negative control (untreated cells). After the treatment period, the cells were washed with PBS twice and fixed in 2% paraformaldehyde for 15 min. Following fixation, the cells were stained with the Hoechst 33,258 solution (Thermo Fisher Scientific). Finally, the stained cells were examined under a confocal laser scanning microscope (Zeiss LSM 510 Meta), to observe and analyze changes in nuclear morphology. Tests were conducted in triplicate.

### 2.8 Cell proliferation

SK-MEL-28 cells were seeded in 96-well plates at a density of 1 × 10^4^ cells per well. After allowing the cells to incubate for 24 h, cells were treated with native (12.5 μg/mL) or detoxified CTX (50 μg/mL) for 24 h. Cells treated with culture medium were used as negative control (untreated cells). Subsequently, the Cell-Light EdU DNA Cell Proliferation Kit (RiboBio) was used, following the manufacturer’s instructions, to analyze the cell proliferation rates. The results were expressed as a percentage (%) of mitotic nuclei. Tests were conducted in triplicate.

### 2.9 Cell cycle analysis

To assess cell cycle progression, 3.5 × 10^5^ SK-MEL-28 cells were seeded in 6-well plates. After a 12-h period of starvation, cells were treated with native (12.5 μg/mL) or detoxified CTX (50 μg/mL) for 24 h. Cells treated with culture medium were used as negative control (untreated cells). Following treatment, the cells were carefully harvested and fixed in 70% ethanol. Subsequently, the cells were washed and resuspended in 200 μL of labeling solution (91.4 mg of magnesium chloride; 77.4 g of sodium citrate; 47.66 g of Hepes; 10 uL of Triton-X, 0.5 mL of propidium iodide, and 9,490 mL of water). The cell suspension was incubated on ice, protected from light, for a duration of 30 min. The percentages of cells in the G0/G1, S, and G2/M phases were quantified using a flow cytometer (Becton–Dickinson FACScanto II). Data from 10,000 cells were collected in each file. Subsequent analysis of the collected data was conducted using FlowJo v.10 software. The results were expressed as percentage (%) of cells in each phase of the cycle. The experiments were conducted in triplicate.

### 2.10 Cell migration and invasion assays

Cell migration was initially evaluated by wound healing assay. The SK-MEL-28 cells were initially plated on 6-weel plates (3.5 × 10^5^ cells/well). When the cells reached about 90% confluence, a sterile 200 μL pipette tip was used to create a perpendicular wound by scratching the cell monolayer. After creating the wound, the cells were washed to remove any debris and the initial state was photographed using an inverted microscope equipped with a camera (Zeiss Axio Vert A1, 10x). The cells were then treated with native (12.5 μg/mL) or detoxified CTX (50 μg/mL) for 12 h. Cells treated with culture medium were used as negative control (untreated cells). After the treatment, cell migration was photographed. Quantification of cell motility was performed using ImageJ software by measuring the wound width in three randomly selected microscopic fields. The results were expressed as percentage (%) of cell migration, with untreated cells considered 100% viable for reference.

Additionally, migration and invasion were evaluated using Transwell 8.0 µm pore polycarbonate membrane inserts. In brief, 5 × 10^4^ SK-MEL-28 cells in serum-free culture medium were seeded into an uncoated upper chamber for migration assay, or into an upper chamber coated with Matrigel for the invasion assay. The lower chamber was filled with a culture medium containing 10% fetal calf serum. The cells in both the migration and invasion assays were then treated with native or detoxified CTX for 12 h. Cells treated with culture medium were used as negative control (untreated cells). Following the treatment period, migrated and invaded cells were fixed with paraformaldehyde 4%, stained with hematoxylin, and then counted under the light microscope (Zeiss Axio Vert A1, 10x). Tests were conducted in triplicate. The results were expressed as percentage (%) of cell migration or cell invasion, with untreated cells considered 100% viable for reference.

### 2.11 Statistical analysis

Statistical analyses of the data were performed using GraphPad Prism. The data were analyzed using One-Way ANOVA and Two-Way ANOVA. the results were considered statistically significant when the *p*-value was less than 0.05 (*p* < 0.05).

## 3 Results

### 3.1 Detoxified CTX presented reduced phospholipase activity

**FIGURE 1:**
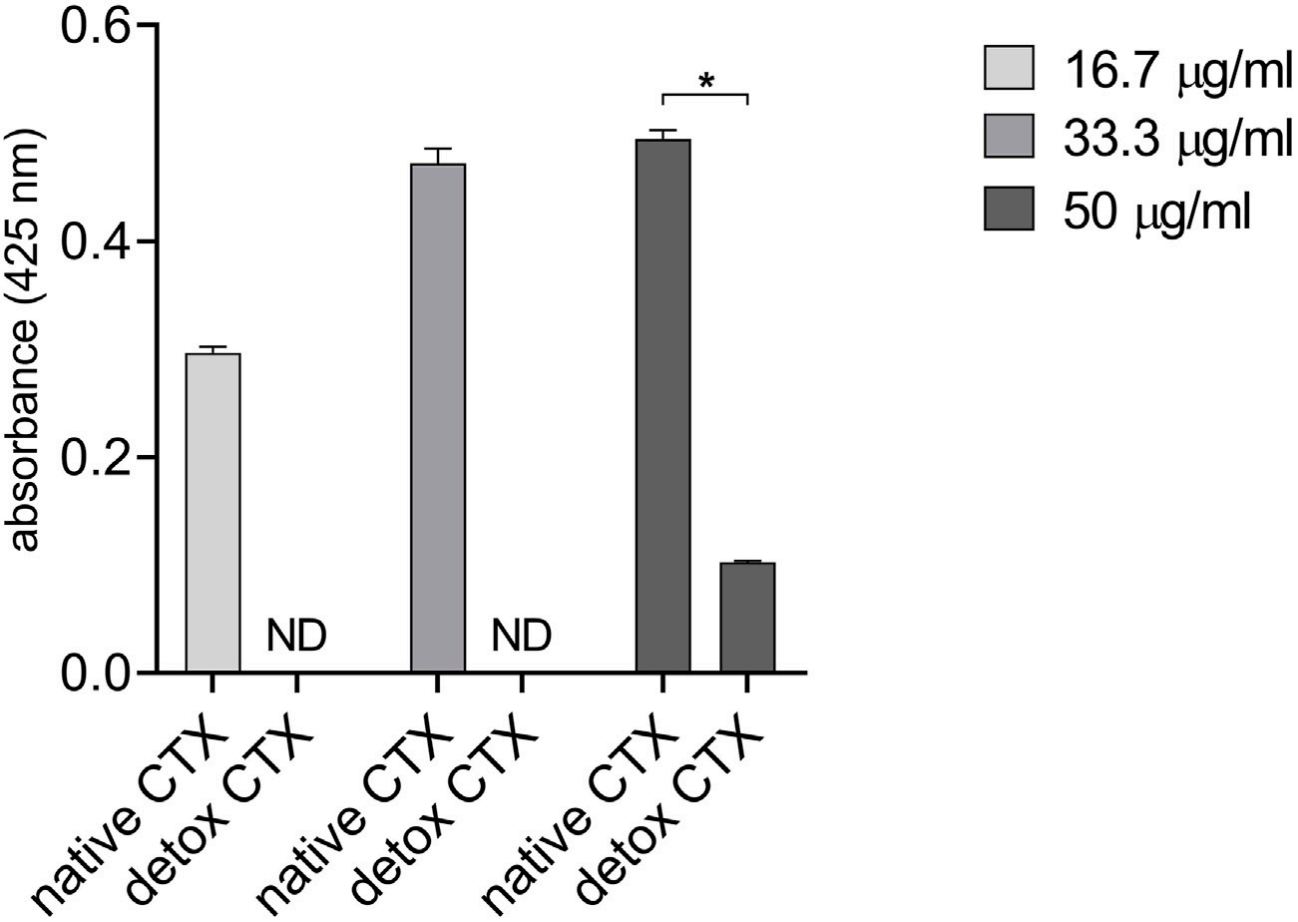
Phospholipase activity of native and detoxified CTX. Data are presented as mean ± SD. ND: not detected. *p < 0.05 between native and detoxified CTX. The experiment was conducted in technical triplicate.

It is established that phospholipases are the primary components responsible for the toxicity and lethality of crotalid venoms (Muller et al., 2018; Baudou et al., 2021). Then, to confirm the detoxification of CTX after autoclaving, we biochemically characterize native and detoxified CTX phospholipase activities. Native CTX presented phospholipase activity at the three concentrations tested, while the analysis of the detox CTX showed that the detoxification process strongly reduced its catalytic activity. Notably, at low concentrations, the phospholipase activity of detoxified CTX was undetectable (Figure 1).

### 3.2 Native and detoxified CTX reduced the viability of melanoma cells selectively

**FIGURE 2:**
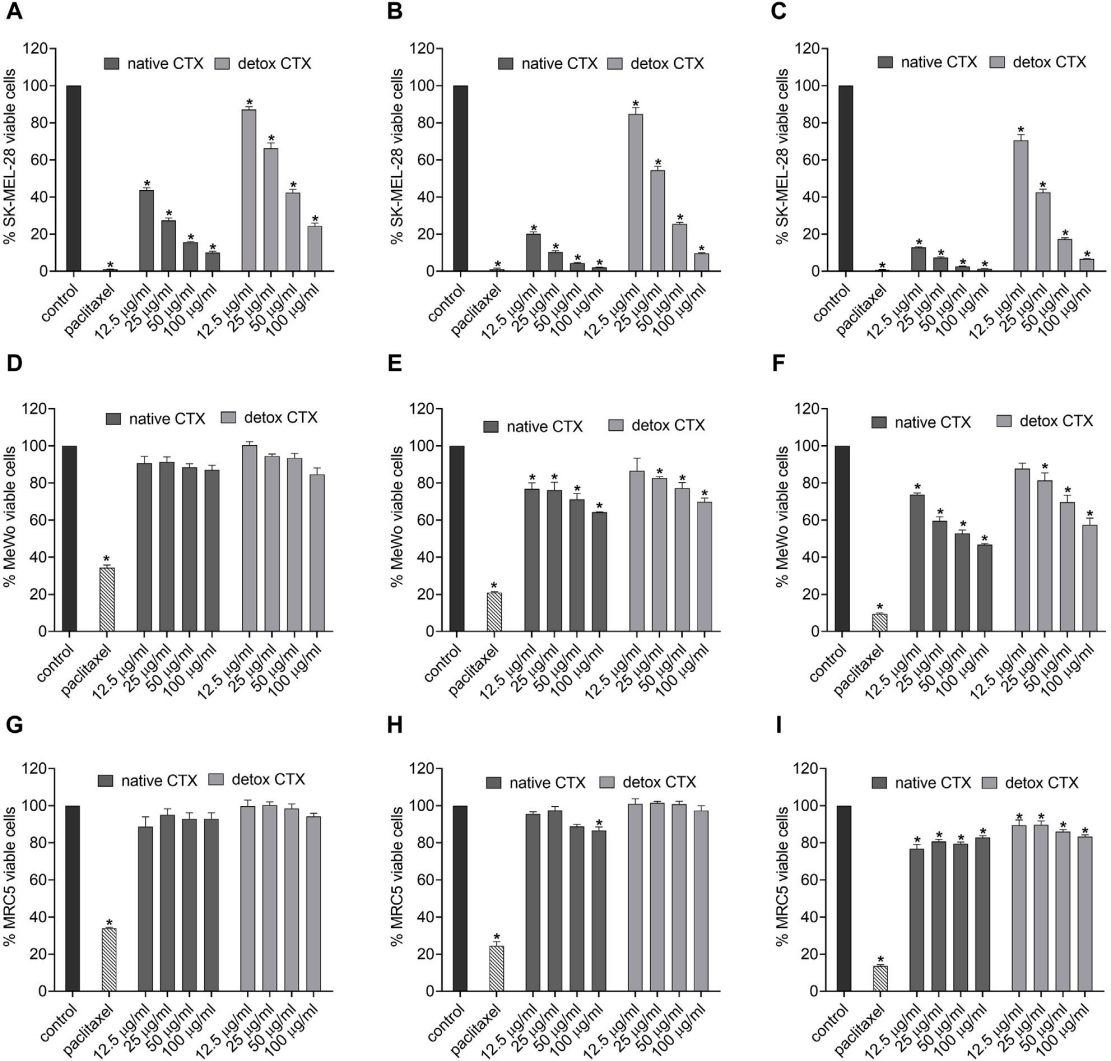
Percentage of viable cells in SK-MEL-28 **(A–C)**, MeWo **(D–F)** and MRC-5 **(G–I)** cell lines after treatment with native or detoxified CTX for 24 **(A, D, G)**, 48 **(B, E, H)**, and 72 h **(C, F, I)**, respectively. Control: untreated cells (cells treated with medium culture). Paclitaxel: 85 μg/mL, positive control. Data are presented as mean ± SD. *p < 0.05 compared to control. The experiments were conducted in technical triplicate.

The cytotoxicity assessment was conducted using the MTT assay, which relies on the activity of the mitochondrial enzyme succinate dehydrogenase in viable cells to reduce the 3-(4,5-dimethylazol-2-yl)-2,5-diphenyl tetrazolium bromide salt to formazan. Our results showed that both native and detoxified (Figures 2A–F) CTX significantly and markedly reduced SK-MEL-28 melanoma cell viability in a concentration-dependent manner after 24, 48, and 72 h of incubation. The cytotoxic effects of CTX in MeWo cells was also observed, however, in a lower intensity. In addition, in this cell line the reduction in cell viability was detected only after 48 and 72 h of treatment for all tested concentration of native CTX and from 25 μg/mL detoxified CTX. In addition, it is worth noting that the cytotoxic effect observed for the detoxified form was less intense than that observed for the native form. Importantly, these cytotoxic effects were found to be selective to melanoma cells, as the viability of fibroblasts was less affected by native or detoxified CTX (Figures 2D–F). Given these results, we conducted the further experiments only with the SK-MEL-28 cell line due to its better response to CTX treatments. Also, the concentrations of 12.5 μg/mL of CTX and 50 μg/mL of detoxified CTX, and the time of 24 h, were chosen for subsequent assays. This selection was made because, under these conditions, approximately 40% of the cells remained viable, which allows for the study of mechanisms of action in the cell death process.

### 3.3 Native and detoxified CTX-induced cell death

**FIGURE 3:**
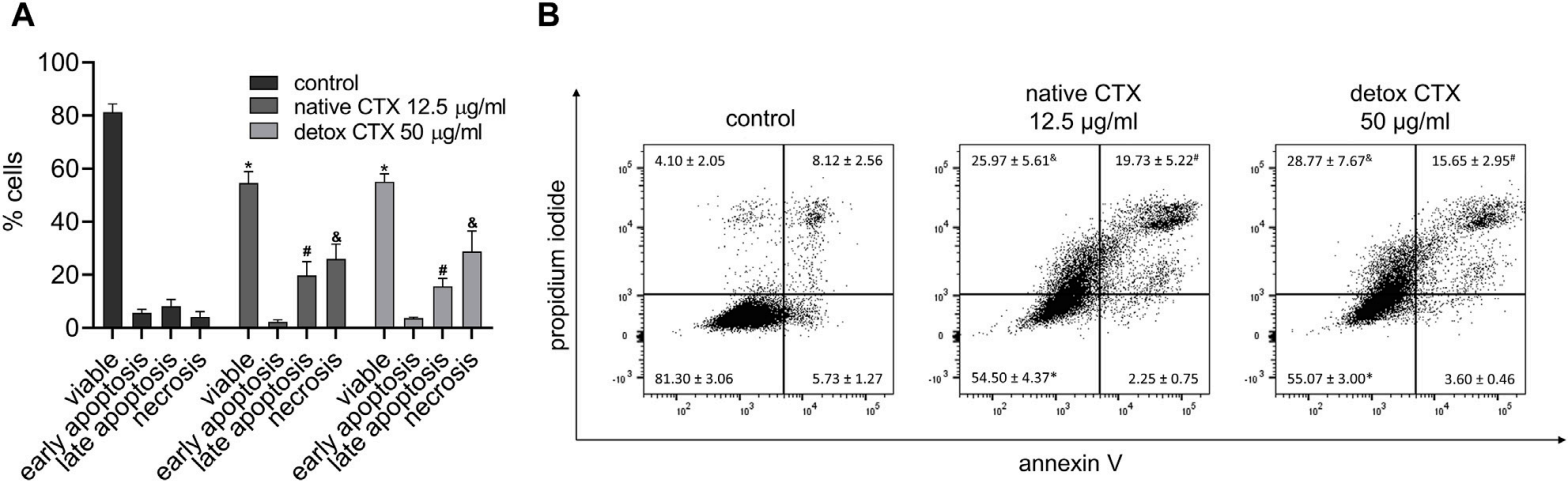
Quantification **(A)** and representative dot plots **(B)** of viability, early apoptosis, late apoptosis, and necrosis rates (%) in SK-MEL-28 cells treated with native or detoxified CTX for 24 h. Control: untreated cells (cells treated with medium culture). Data are presented as mean ± SD. *p < 0.05 compared to live control cells, #*p* < 0.05 compared to late apoptosis in control cells, and *p* < 0.05 compared to necrosis in control cells. The experiment was conducted in technical triplicate.

To gain insight into the death pathway induced by native and detoxified CTX, a quantitative analysis of cellular apoptosis and necrosis rates was carried out using flow cytometry with annexin V-FITC and propidium iodide dual-labeling. The results demonstrated that both CTX forms led to increased percentages of necrosis and late apoptosis in SK-MEL-28 melanoma cells (Figure 3).

### 3.4 Native and detoxified CTX-induced activation of caspases-3/7 and formation of apoptotic bodies

**FIGURE 4:**
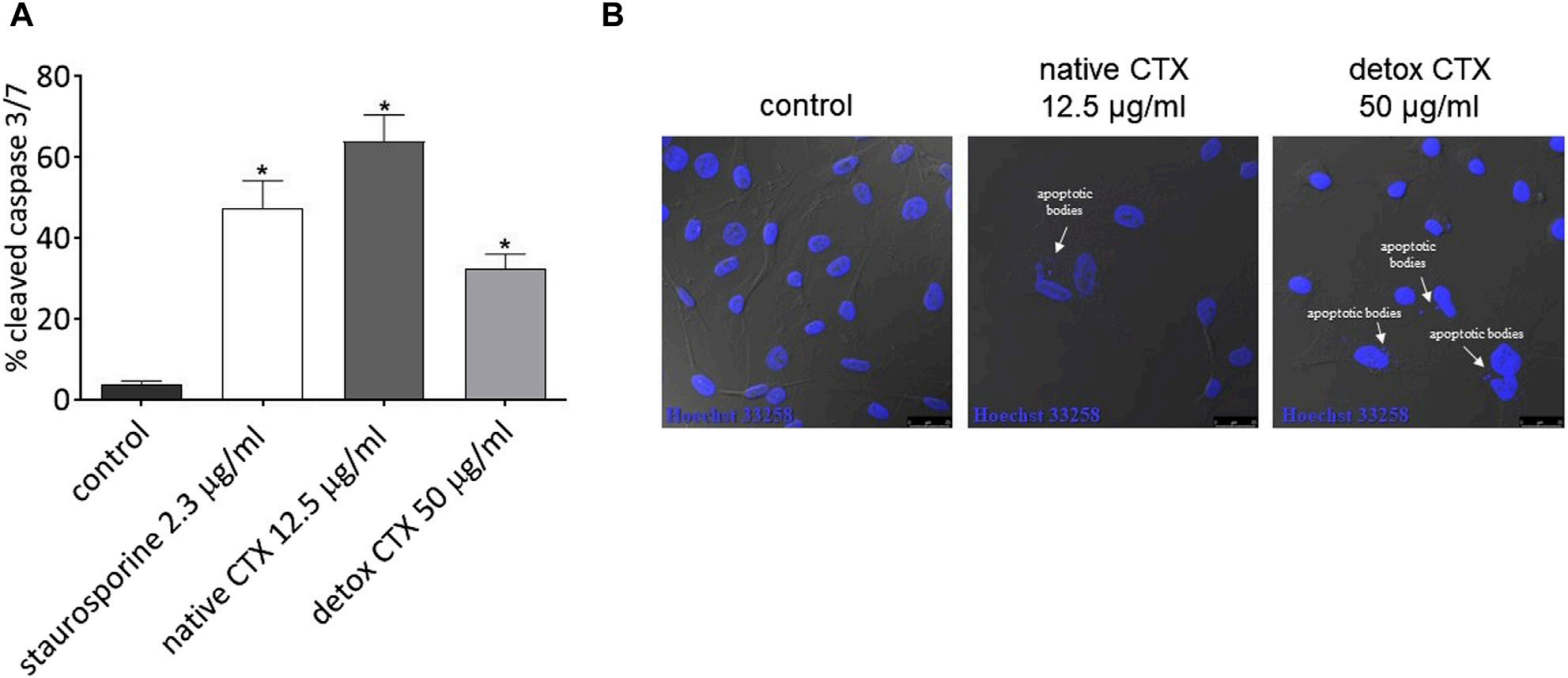
**(A)** Percentage of cleaved caspases-3/7 in SK-MEL-28 cells after treatment with native or detoxified CTX for 24 h. Control: untreated cells. Staurosporine: positive control. Data are presented as mean ± SD. *p < 0.05 compared to control. **(B)** Formation of apoptotic bodies in SK-MEL-28 cells after treatment with native or detoxified CTX for 24 h. Control: untreated cells (cells treated with medium culture). The experiments were conducted in technical triplicate.

Subsequently, to confirm the cell death by apoptosis, the effect of native and detoxified CTX on caspase activity in SK-MEL-28 melanoma cells was evaluated. HCS analysis showed that treatment with native and detoxified CTX resulted in the increased activity of cleaved caspase-3/7 (Figure 4A). Additionally, through confocal microscopy, the formation of apoptotic bodies was observed following treatment with both native and detoxified CTX (Figure 4B).

### 3.5 Native and detoxified CTX reduced cell proliferation

**FIGURE 5:**
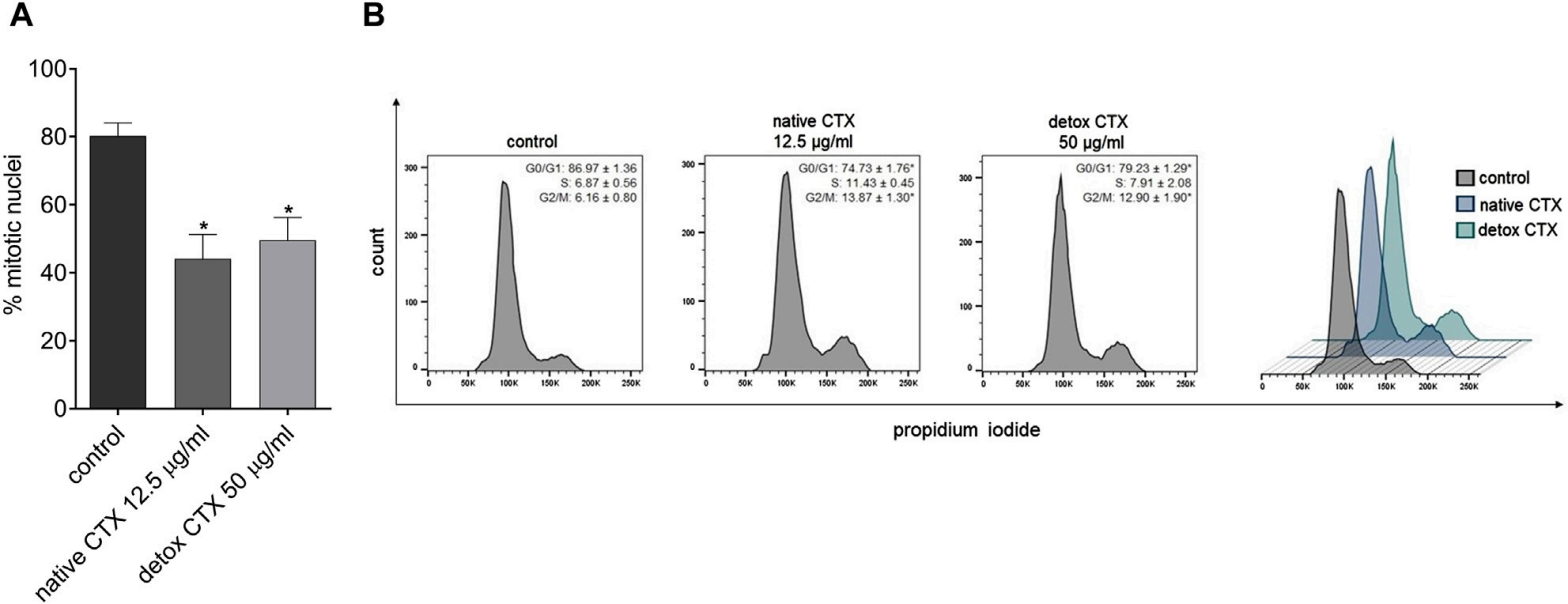
**(A)** Percentage of SK-MEL-28 cell proliferation after treatment with native and detoxified CTX for 24 h. Data are presented as mean ± SD. *p < 0.05 compared to control. **(B)** Representative histograms and percentage of SK-MEL-28 cells in each phase of the cell cycle after treatment with native and detoxified CTX for 24 h. Control: untreated cells (cells treated with medium culture). Data are presented as mean ± SD. *p < 0.05 compared to control. The experiments were conducted in technical triplicate.

Further, the antiproliferative effect of native and detoxified CTX was evaluated by EdU incorporation assay. The kit is designed to label newly synthesized DNA, and it involves the incorporation of EdU (5-ethynyl-2′-deoxyuridine) into replicating DNA during cell division. The findings revealed that both forms of CTX significantly reduced SK-MEL-28 cell proliferation (Figure 5A).

### 3.6 Native and detoxified CTX-induced cell cycle at the G2/M phase

To assess the mechanisms of antiproliferative CTX effects, the progression of cell cycle analysis was conducted. The results revealed that treatment with both native and detoxified CTX led to a substantial reduction in the number of cells in the G0/G1 phase of the cell cycle. Concurrently, there was an increase in the number of cells in the G2/M phase (Figure 5B). This suggests that treatment with CTX disrupts normal cell cycle progression, leading to cell cycle arrest at the G2/M phase, which can contribute to the inhibition of SK-MEL-28 cell proliferation.

### 3.7 Native and detoxified CTX reduced cell migration and invasion

**FIGURE 6:**
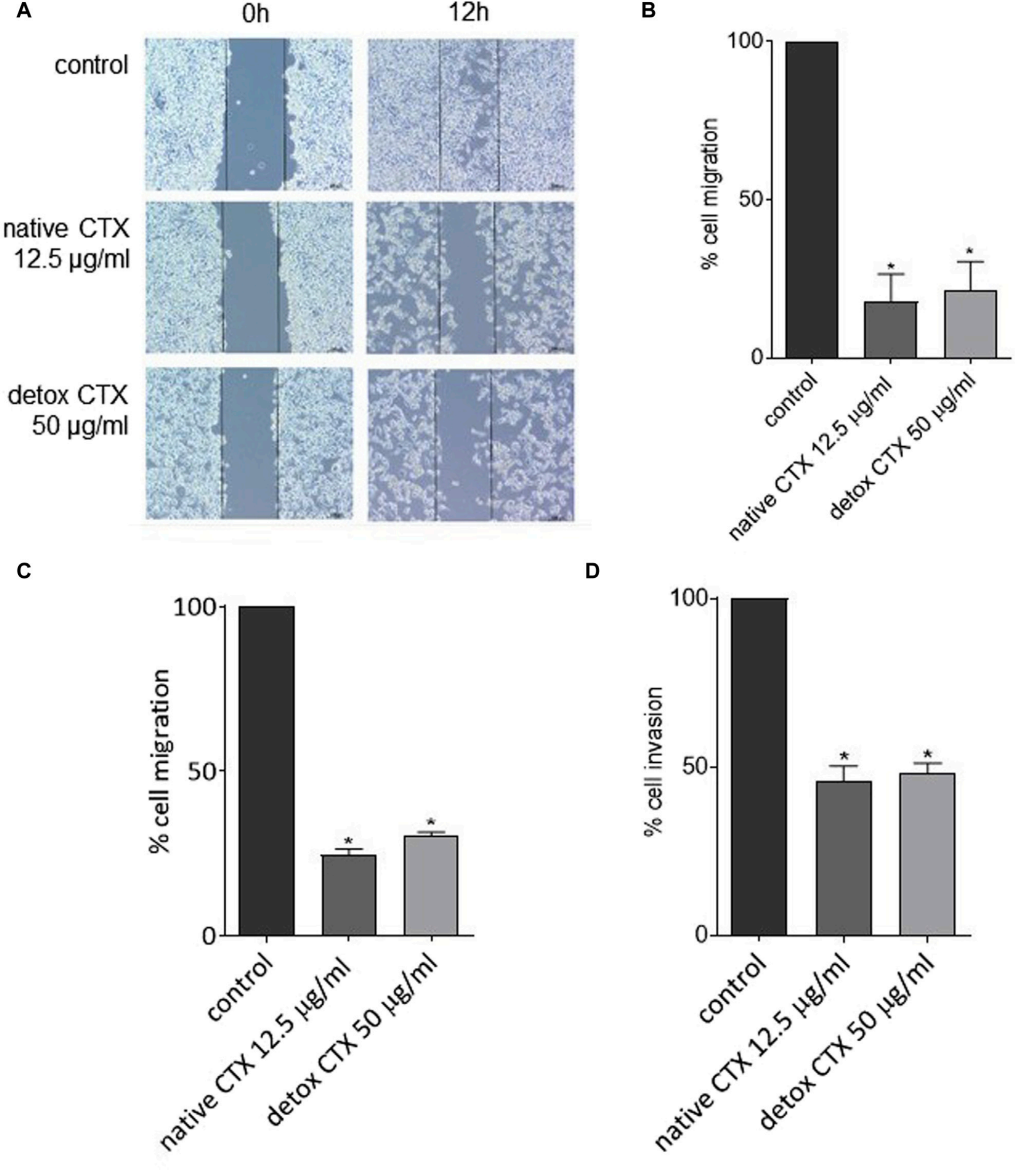
Representative images **(A)** and percentages **(B)** of SK-MEL-28 cell migration after treatment with native or detoxified CTX for 24 h evaluated by wound healing assay. Percentages of cell migration **(C)** and invasion **(D)** of SK-MEL-28 cells after treatment with native or detoxified CTX for 24 h evaluated by transwell assay. Control: untreated cells (cells treated with medium culture). Data are presented as mean ± SD. *p < 0.05 compared to control. The experiments were conducted in technical triplicate.

Native and detoxified CTX were also evaluated regarding their effects on migration and invasion of SK-MEL-28 cells. Using two complementary methods, wound healing and Transwell assay, we found that native and detoxified CTX treatments reduced cell migration, as evident from the decrease in cell gap closure (Figures 6A, B) and the diminished number of migrated cells through the Transwell membrane (Figure 6C). Furthermore, treatment with native and detoxified CTX resulted in a decreased invasion of SK-MEL-28 cells through Matrigel (Figure 6D).

## 4 Discussion

Animal venoms are intricate mixtures of compounds, containing proteins, peptides, biogenic amines, enzymes, and neurological growth factors. These components interact with various targets, such as ionic channels, receptors, enzymes, and membrane transporters. Apart from the tissue damage induced by most of the venom, several drugs derived from their components have been clinically used for several diseases, such as captopril, derived from snake venom and used as an antihypertensive; ziconotide, derived from cone snails and used for chronic pain; and exenatide, derived from lizards and used for type 2 diabetes mellitus control. Nevertheless, in certain cases, the potential therapeutic benefits of animal venom components are hindered by their toxicity, especially in the context of debilitating chronic diseases (Zambelli et al., 2016; Bordon et al., 2020).

Crotoxin (CTX), the primary component found in the venom of the *C. durissus terrificus* snake, possesses various therapeutic properties. However, in high doses it can be lethal due to its neurotoxic, myotoxic, and paralyzing effects (Cañas, 2022; Hayashi et al., 2022). In this study, we explored the antitumor potential of a detoxified form of CTX and its mechanism of action on human melanoma cells. Our findings demonstrated that both native and detoxified CTX led to a reduction in melanoma cell viability. Previously, some studies attributed the antitumor effects of CTX to its phospholipase activity (Corin et al., 1993; Donato et al., 1996). Our investigation revealed that even though the detoxified version of CTX exhibited a decrease in phospholipase activity compared to native CTX, it retained its cytotoxic activity. Specifically, at a concentration of 50 μg/mL, the phospholipase activity of detoxified CTX was approximately 4.8 times lower than that of native CTX. In contrast, the reduction in cytotoxic activity was only roughly twice as pronounced within a 24-h treatment period for SK-MEL-28 cells. For MeWo cells, the difference in cytotoxicity between native and detox CTX is even less pronounced. Besides that, also at the concentration of 25 μg/mL, detoxified CTX induces cytotoxicity in both SK-MEL and MeWo cell lines, with this concentration falling within the range where no phospholipase activity is observed (16.7 and 33.3 μg/mL). These data suggest that the phospholipase activity may be important but not essential for the cytotoxic activity of CTX. Other components of the toxin may also be associated with its antitumor properties. Indeed, studies on the antitumoral effects of snake venom phospholipases have indicated that mutations at codon 49 (Asp > Lys) can result in the loss of enzymatic activity. In such cases, the antiproliferative effects were linked to the C-terminal region of phospholipase A2 (Araya and Lomonte, 2007; Costa et al., 2008; Gebrim et al., 2009).

Importantly, both native and detoxified CTX exhibited remarkable selectivity towards melanoma cells, mainly for SK-MEL-28 cells, as the cytotoxic effects on non-tumoral cells were less pronounced. Previous research has shown that cells with elevated epidermal growth factor receptor (EGFR) expression tend to be more sensitive to CTX effects (Donato et al., 1996). This observation may elucidate our findings, as the MRC-5 cells, with their low EGFR expression, displayed reduced sensitivity (Song et al., 2018; Liu et al., 2019). Another plausible explanation for this selectivity is rooted in the strong affinity of CTX for interacting with anionic lipid model membrane (de Araújo Pimenta et al., 2021) and it is well-established that cancer cells often overexpress anionic molecules on their membranes (Le et al., 2019).

Regarding the mechanism of cell death, our investigation revealed elevated levels of necrosis and late-stage apoptosis in melanoma cells treated with both native and detoxified CTX when compared to untreated cells. Notably, the SK-MEL-28 melanoma cell line carries a mutation in the *TP53* gene, which typically results in greater resistance to apoptosis, as p53 is a central inducer of apoptosis in response to stress signals (Kastenhuber and Lowe, 2017). This may explain the higher levels of necrosis observed after treatment with both native and detoxified CTX. However, it is important to emphasize that the induction of apoptosis does not rely solely on a single p53-dependent pathway (Ashkenazi, 2008). To further validate the occurrence of apoptosis induced by native and detoxified CTX, the levels of active caspase-3 and caspase-7 were quantified following treatment. Caspase-3 and caspase-7 are universally activated during apoptosis, irrespective of the initial stimulus, and both act as effectors of this type of cell death. They cleave a diverse array of protein substrates, leading to the morphological and biochemical changes typically observed in apoptotic cells (Elmore, 2007). Our results demonstrated that native and detoxified CTX increased the activity of caspases-3/7 in melanoma cells. Additionally, we observed the presence of apoptotic bodies following treatment, providing further confirmation of apoptosis induction by CTX in both forms. Importantly, although our results have demonstrated cell death through necrosis and apoptosis, other mechanisms may be occurring. Indeed, some authors have liked the occurrence of autophagy with the reduction of tumor cell viability after treatment with CTX (Yan et al., 2006; Yan et al., 2007; Han et al., 2014), which cannot be excluded.

Furthermore, in our cell cycle analysis, we observed that both native and detoxified CTX induced a cell cycle arrest at the G2/M phase, resulting in the inhibition of cell proliferation. A similar result was previously found in breast cancer cells and the G2/M cell cycle arrest caused by CTX was accompanied by p-ERK1/2 inhibition (Almeida et al., 2021). Alongside the induction of necrosis and apoptosis, this G2/M cell cycle arrest represents another critical mechanism triggered by both native and detoxified CTX to impede tumor development.

It is important to point out that several isoforms of CTX were identified in the *C. durissus terrificus* venom, formed by random association of various combinations of CA or CB subunit variants (Faure and Bon, 1987; Faure and Bon, 1988). These diverse isoforms slightly but significantly differ in their enzymatic and toxicological effects (Faure et al., 1993). In this study, the complete CTX was used, without the determination or characterization of possible isoforms, and it was purified from the crude venom resulted from a pool of *C. durissus terrificus* snakes. Therefore, we cannot confirm whether different isoforms exhibit varying cytotoxic effects in melanoma cells, and it may be a promising approach for further better understanding their effects. Additionally, to the best of our knowledge, differences in the antitumoral effects of CTX induced by various isoforms have not yet been evaluated.

In the context of melanoma, the occurrence of metastasis results in poor prognostic, with a median survival period of 9 months and a long-term survival rate of just 10% (Turner et al., 2018). Metastasis is a complex multistep process that involving the migration and invasion of cancer cells (Tahtamouni et al., 2019). Therefore, the quest for drugs capable of inhibiting these cellular mechanisms in melanoma is of paramount importance. We found that native and detoxified CTX reduced migration and invasion of melanoma cells, thus contributing to the suppression of their metastatic potential. In line with our findings, previous studies on oral cancer cells have also reported the inhibitory effects of CTX on crucial modulators associated with cell migration and invasion, including MMP-2, MMP-9, and COL1A1 (da Rocha et al., 2023).

## 5 Conclusion

In summary, our collected data lead us to the conclusion that both native and detoxified forms of CTX exhibit a potent antitumor effect on human melanoma cells. This effect is attributed to their cytotoxic activity, resulting in the induction of apoptosis and necrosis in melanoma cells. Furthermore, both native and detoxified CTX induce cell cycle arrest, consequently impeding the proliferation of melanoma cells. Notably, these CTX forms also inhibit the mechanisms of cell migration and invasion. Consequently, our findings provide robust evidence of CTX’s antitumor potential. Importantly, the discovery that a detoxified version of CTX retains antitumor effects is particularly promising. Since several toxic effects of CTX are attributed to its phospholipase activity, the use of the detoxified CTX suggests a possible improvement in the safety issues. In addition, this encourages further investigations aimed at identifying specific components of the molecule that exhibit activity against tumor cells while avoiding significant toxic side effects. Identifying smaller fragments could lead to creating new molecules through chemical synthesis, bypassing the issue of low yield when using natural compounds. In summary, this research could lead to safer and more effective antitumor treatments based on CTX.

## Data Availability

The original contributions presented in the study are included in the article/Supplementary Material, further inquiries can be directed to the corresponding author.
